# Probationer Nurses Who Need a Champion

**Published:** 1918-10-12

**Authors:** 


### SOME LONDON NURSES IN TRAINING.

### Probationer Nurses who Need a Champion.

The following statement, which we are assured is correct, deals with the case of the probationer nurses and defines their circumstances and position :—

Probationers have noticed in the article on the London Hospital correspondence the remark that no complaint had been made by the probationer nurse. Why? I can tell you. Simply from fear. Many of our nurses are dependent for their bread on their training, and having finished a certain time they live in daily fear of being sent away from hospital. If this is not so, it is because, having done what they think to be the hardest part of their training, they wish to get their certificate. According to our agreement we may be sent away without any reason being given, and we have no redress. Feeling is running very high just now at the "London," although those in authority choose to disregard it. The probationers and nurses are only waiting for a lead, most of us being too tired to possess any initiative ourselves. We have just passed through a very heavy time—no less than 130 nurses being off duty either on holidays or sick, because influenza and pneumonia have been very rife amongst us, nurses keeping on duty long after they should have been off sick. As an example the sister who died recently from pneumonia was on duty two days with a temperature of over 100°, and it was known by one of matron's assistants.

All of us would be only too pleased to have a three years' training as a probationer, and we feel we are still untrained at the end of two years. A nurse now on the private staff told me the other day that she has only been

**Some London Nurses in Training—**(*continued*).

in an operating theatre once, and she has been in hospital three years. A probationer does theatre work or not just as it happens, and yet we are trained surgical nurses!

It frequently happens that a nurse finishes her classes and takes her final examination before she has been in hospital ten months. What can she know of her work, which till then has been that of a common drudge, sweeping, dusting, cleaning sinks? The poor girl is too dead tired in her first year to have any other aim than to get finished in time.

On night duty the probationer, after having been on duty from 9.20 P.M. to 9.20 A.M., taking all her meals, if she has time for them, in the ward, has her dinner at 10 A.M., and then is expected to attend a class from 10.30 A.M. to 11.30 A.M. twice a week, and has to get up from her bed an hour earlier twice a week to attend a lecture and another class. Tell me, would any man work like this?

Will nobody take up the cause of these nurses?

